# Crane-powered pectus excavatum repair: the NeoPectus surgery

**DOI:** 10.3389/fsurg.2023.1267009

**Published:** 2023-12-19

**Authors:** Hyung Joo Park, Gongmin Rim, Seung Keun Yoon

**Affiliations:** ^1^ Department of Cardiothoracic Surgery, Nanoori Hospital, Seoul, Republic of Korea; ^2^ Department of Cardiothoracic Surgery, Seoul St. Mary's Hospital, The Catholic University of Korea, Seoul, Republic of Korea

**Keywords:** pectus excavatum, crane, crane-powered approach, sternal screw, sternal pre-lifting

## Abstract

**Introduction:**

The conventional technique for pectus excavatum repair was pushing up the depressed chest wall by turning over the convexity of the bent pectus bar. We developed a new concept in which a total crane lift was used as the source of power to elevate the depressed sternum without using pectus bar leverage. This study aims to verify the efficacy of exclusively crane-powered pectus excavatum repair in recent years.

**Methods:**

Among the 3622 pectus deformity repairs since 1999, 691 cases repaired with the total crane power between 2017 and 2022 were enrolled. The mean age was 12.0 years (3–45). The operative technique involved wire/screw crane elevation of the sternum, the entire chest wall remodeling with the cross or parallel bars, the bridge/claw bar fixations, and other adjunctive techniques. Outcome analysis included morphological variations, patterns of pectus bar placement, and complication rates.

**Results:**

The crane technique and pectoscopy (100%) were used. The bar placements were parallel (22.0%) and cross-bar (47.5%). The bar fixations were the claw fixators for a single bar (30.5%) and the bridge plates for multiple bars (69.5%). The flare-buster and magic strings were liberally used. No serious complications or catastrophic events occurred, but minor complications occurred in 16.9%: pneumothorax in 7.4% (51), pleural effusion in 1.6% (11), and wound problems in 0.4% (3). There was no case of bar displacement.

**Discussion:**

The crane-powered pectus excavatum repair showed excellent results with minimal complications and no bar displacement. This innovative approach, part of the NeoPectus surgery, represents a significant advancement in correcting pectus excavatum deformities by utilizing a crane machine to elevate the chest wall.

## Introduction

The Nuss procedure, an initially groundbreaking approach for pectus excavatum correction, has faced significant challenges due to serious complications, limiting its widespread success (1). These complications include cardiac injury, improper pectus bar positioning, and inadequate chest wall elevation, posing risks to patients (2, 3). This article explores the primary issues contributing to these challenges and introduces the “Crane-powered Pectus Surgery” as an innovative solution.

### Visual obstruction and cardiac perforation

**Figure 1:**
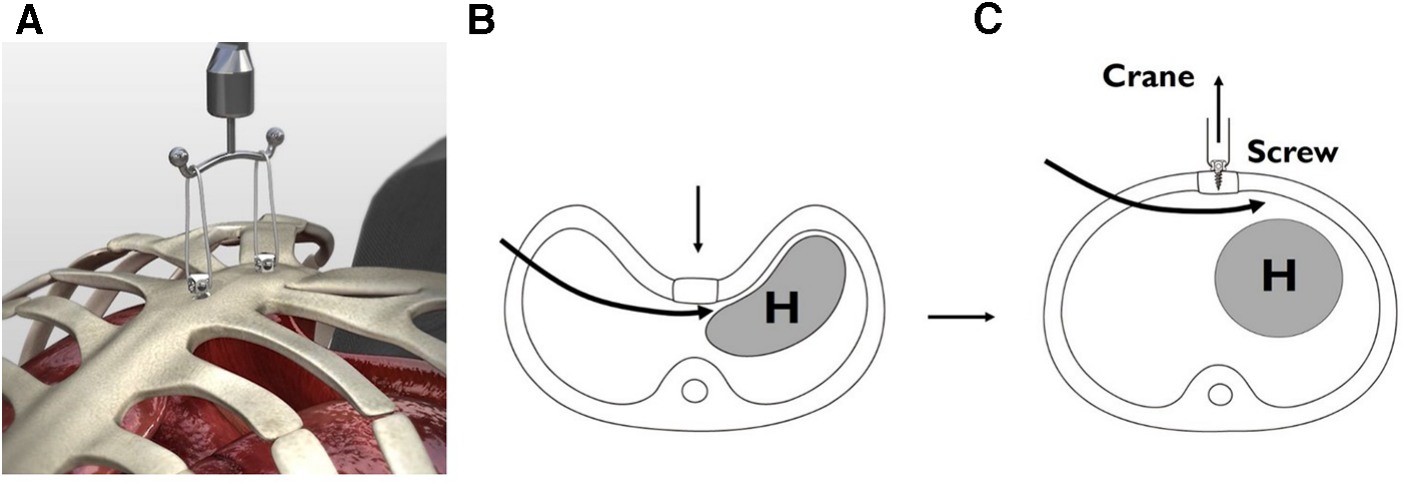
Crane–assisted sternal pre-lifting procedure for depressed sternum (2002 – Present). (**A**) Crane-assisted sternal pre-lifting with multiple screws. (**B**) Before sternal lifting: tight interface between the chest wall and the heart. (**C**) After sternal lifting with screw crane: elevation of the depressed chest wall to normal level, resulting in a wide-open mediastinum.

The Nuss procedure encounters difficulty due to limited visual access caused by the obstructive nature of the depressed chest wall. This tight abutment of the chest wall and heart hinders visualization during surgery, potentially leading to blind or imprecise introducer advancement and subsequent cardiac perforation (Figure 1).

### Inadequate force for chest wall elevation

Insufficient force exerted by the pectus bar turnover, the primary mechanism for elevating the chest wall, becomes problematic, especially in cases with heavy chest walls. This can damage hinge structures, such as intercostal muscles stripping (4).

### The crane elevation

To address these challenges, the author introduced the crane elevation technique in 2002. Initially used as a rescue measure in difficult cases, this method involved using a wire sternal stitch connected to an external strut to lift the sternum, providing additional force for chest wall elevation beyond the standard Nuss approach (5) (Figure 1).

### The crane-powered pectus surgery

**Figure 2:**
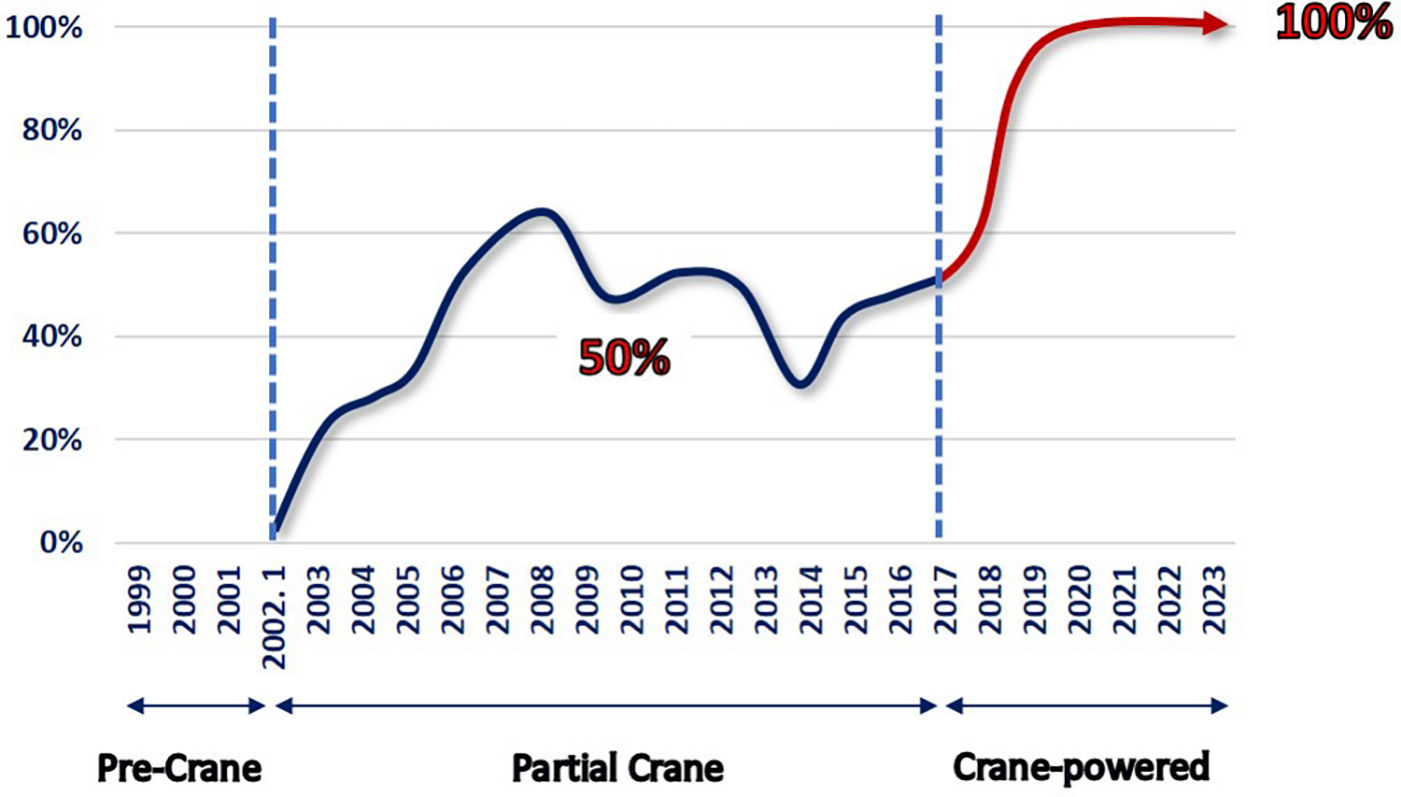
Crane application by the year (1999 –2023). The first crane application occurred in 2002. Since 2017, the total crane strategy involving sternal over-lifting was adopted. In 2019, the crane-powered pectus excavatum repair approach was established. The totally craned approach for every patient was implemented, with a total of 691 patients aged between 3 and 45 years.

Building upon the crane elevation's success, the “Crane-powered Pectus Surgery” was developed in 2017 (6) (Figure 2). This novel approach consistently applies crane elevation to all patients undergoing pectus correction, regardless of age or condition severity. By lifting the sternum beyond the level of the normal chest wall, this technique enhances visibility during the procedure and ensures a more effective correction of the pectus excavatum deformity.

## Material and methods

### Patient cohort

Among 3,622 pectus deformity repairs since 1999, 691 cases repaired with the total crane power between 2017 and 2022 were enrolled in this retrospective study. Demographic data, including age and gender distribution, were collected and analyzed. Morphological assessment with asymmetry including Grand Canyon subtypes were assessed. Complications related to the crane-powered pectus excavatum repair procedure were documented and analyzed to evaluate the safety profile of the technique.

### Operative steps for crane-powered pectus excavatum repair

1. **Crane Pre- Elevation of the Sternum**: The initial step of the repair procedure involves elevating the sternum using wire suturing or sternal crane screws. A table-mount crane system, such as the Easy Crane, is utilized to lift the sternal wire or screw to the maximum level. Multiple wires or screws may be used to ensure complete elevation of the entire anterior chest wall or to support heavy chest walls with double or triple support.
2. **Pectus Bar Introduction**: The pectus bar(s) is shaped using the terrain contour matching (TERCOM) scheme (7). A pectoscope (Primemed, Seoul, South Korea) is introduced for direct vision inside the chest cavity and to guide the placement of the pectus bar(s). The pectoscope is designed for thoracoscopy with integrated dissection capabilities. This unique device features a curved shaft tailored to seamlessly navigate beneath the concave chest wall following its contour. Equipped with a tactile view interface, it facilitates precise visual guidance when traversing the closely juxtaposed juncture of the pericardium and chest wall. Consequently, we maintain continuous visibility along our designated route, ensuring a secure and controlled procedure. This approach obviates the necessity for additional introduction instruments, streamlining the process.

Since the chest wall is already elevated using the crane, the pectus bar(s) can be accurately positioned and turned over without resistance from the chest wall. The pectus bars are not pushing up the chest wall, but are placed beneath the chest wall that has already been raised. Subsequently, the chest wall is gently lowered onto the prepositioned pectus bars by releasing the crane lifting.

3. **Pectus Bar Stabilization**: All the set pectus bars are linked together with a single bridge plate bilaterally, creating a rock-solid structure to prevent any shaking or displacement. This stabilization procedure is performed with full crane elevation, ensuring the easiest and safest execution of the process (8).
4. **Final Touches (Chest Wall Ironing)**: After elevating the depressed chest wall for repair of pectus excavatum, it is common to observe lower costal flare. To address the flared costal cartilages and achieve a smooth chest wall, the flare-buster sandwich technique is applied. For focal protuberances in the para sterna area after correcting the excavatum, liberal application of the magic strings is done (9).

In our study, we observed the presence of lower costal flares in all enrolled patients. While there was some variability in the severity of these flares, we noted significant improvements in the chest wall contour, approaching a more normal anatomical appearance, with the implementation of the flare-buster technique. Given the inherent scaphoid nature of the chest wall, we expected some degree of flaring in pectus excavatum deformities.

Initially, we primarily employed the flare-buster technique in younger patients with pronounced flaring. However, as our experience grew, we began to apply this technique to all patients, including adults with more resistant chest wall flares. To address these challenging cases, we introduced a compressor machine to effectively reduce the resistant flares and achieve a flat contour. Consequently, we now routinely incorporate the flare-buster technique in all of our chest wall repair procedures.

In contrast, we conducted thorough investigations to address focal parasternal protuberances. To tackle this specific issue, we employed the modified sandwich technique, which is referred to as the “magic string” technique. Our goal was to achieve a flat chest wall appearance in cases where these protuberances were present.

## Results

A total of 691 patients who underwent crane-powered pectus excavatum repair were included in this study, with an age range of 3 to 45 years and a mean age of 12.0 years. The male-to-female ratio was 4.9:1.

Assessment of morphological types revealed that 50% (345) of patients had asymmetric pectus excavatum, with 20.7% (143) falling under the Grand Canyon subtypes (10, 11). The use of pectus bars was distributed as follows: single bar in 30.5% (211) of cases, and multiple bars in 69.5% (480) of cases. Types of bar placement included single bar in 30.5% (211) of cases, parallel bars in 22.0% (152) of cases, and cross bars or cross plus horizontal bars (XI) in 47.5% (328) of cases (12, 13). Pectus bar stabilization techniques involved the use of a claw fixators for single bar cases in 30.5% (211) and bridge stabilization for multiple bars in 69.5% (480) of cases (14, 15). Flare-busters were applied in all patients, and the magic string technique was utilized in 52.3% (362) of cases F.

The overall complication rate was 16.9% (106), but major complications such as bar dislocation or bleeding did not occur. Other minor complications included transient pneumothorax in 7.4% (51) of cases, pleural effusion in 1.6% (11) of cases, and wound problems in 0.4% (3) of cases. Pleural effusion and wound infections were classified as Clavien-Dindo Grade II conditions that could be managed pharmacologically, but pneumothorax needed Grade IIIa intervention with a small-bore catheter drainage of the pleural space (16).

## Discussion

### Neopectus surgery: a novel approach for comprehensive pectus excavatum repair

Since 2017, the author has developed a new approach called “NeoPectus Surgery,” which consists of two unique components: crane-powered pectus excavatum repair (pre-levo surgery) and entire chest wall remodeling to normal anatomy (normo-pectus surgery). NeoPectus surgery started as a novel approach for comprehensive pectus excavatum repair, aimed at achieving complete chest wall remodeling to restore a physiologically and anatomically normal chest wall—the “crane-powered entire chest wall remodeling.” This paradigm shift eliminates the reliance on the pectus bar as the primary force for elevation, and covering the entire chest wall and addressing all present morphological elements of deformity, making it a potential revolutionary technique for comprehensive pectus excavatum repair.

### Evolution of crane-powered pectus excavatum repair

The author introduced the exclusively crane-powered pectus excavatum repair approach in 2019, building upon earlier successes with partial chest wall lifting using the crane technique in selective cases, especially those with challenging heavy chest walls, dating back to 2002 (2)..

This pre-lifting of the sternum offers several advantages. Firstly, it creates more space between the sternum and the heart, which are tightly compressed in pectus excavatum, allowing for better visualization of mediastinal structures and reducing the risk of cardiac injuries during the insertion of the introducer or pectus bar. Secondly, the procedure becomes easier as the heavy chest wall is already elevated, preventing the pectus bar from exerting pressure on the chest wall and protecting the hinge point from damage due to intercostal muscle stripping (17–20).

**Figure 3:**
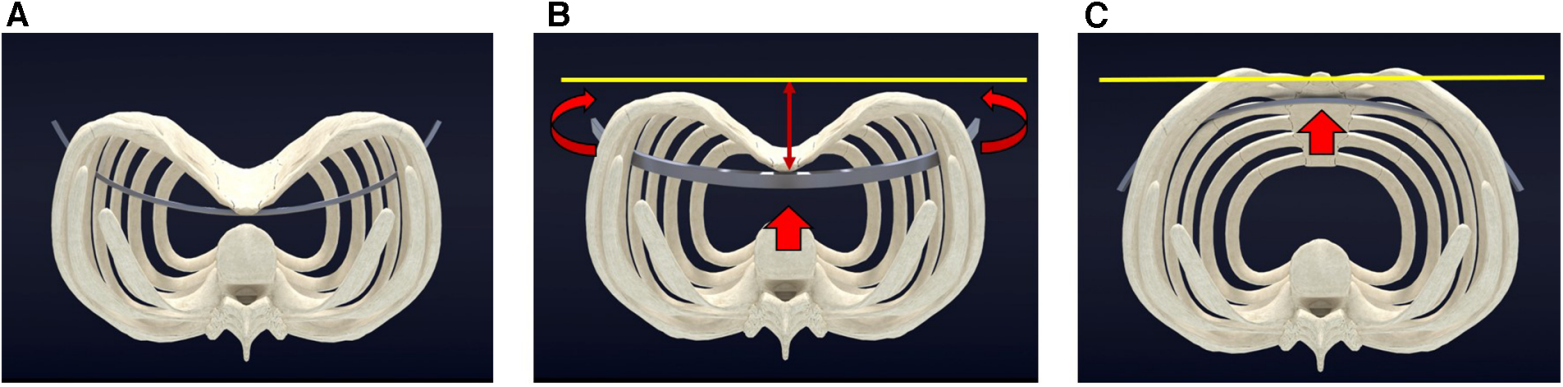
Pectus excavatum repair utilizing pectus bar lever action (Nuss Procedure). (**A**) Pre-operative view of pectus excavatum, prior to sternal elevation, with the pectus bar positioned for turnover. (**B**) During the procedure, the sternum undergoes lifting (indicated by red arrows), as the pectus bar rotates and pushes up the depressed chest wall to reach the desired chest wall height (marked by the yellow line). (**C**) The sternum reaches the target level (yellow line) as the pectus bar completes its full turnover (red arrow).

In the traditional technique pioneered by Donald Nuss, a bent pectus bar was employed to push up the depressed chest wall. However, this approach presented significant problems, impeding safe and effective repair of pectus deformities. The lack of pre-lifting the depressed sternum resulted in limited space between the heart and chest wall, leading to restricted access and an increased risk of cardiac perforation and bleeding. Moreover, the pectus bar, acting as the primary force generator, caused stripping of intercostal muscles and difficulties in accurate positioning or rotation against resistance, ultimately resulting in suboptimal repair or procedural failure (2) (Figure 3).

**Figure 4:**
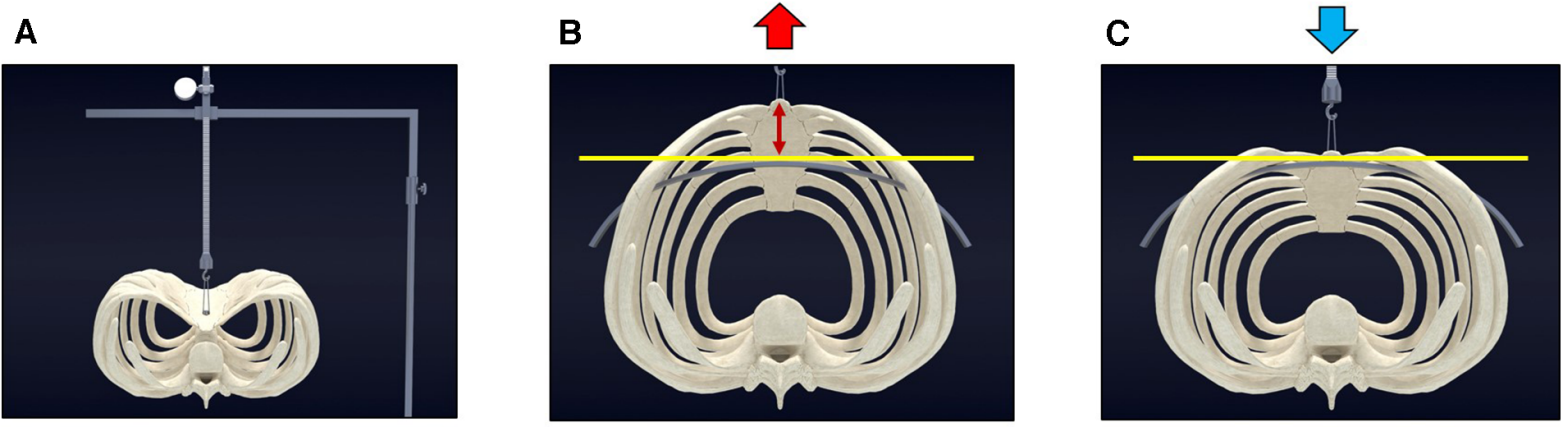
Crane–powered pectus excavatum repair (Park Procedure). (**A**) Pre-operative view of pectus excavatum before sternal elevation. (**B**) The sternum is over-lifted (indicated by red arrows) using the screw crane, elevating it above the targeted chest wall height (marked by the yellow line). The pectus bar can be safely and easily positioned without encountering resistance from the chest wall. (**C**) The sternum is gently set down (blue arrow) onto the accurately positioned pectus bar.

**Figure 5:**
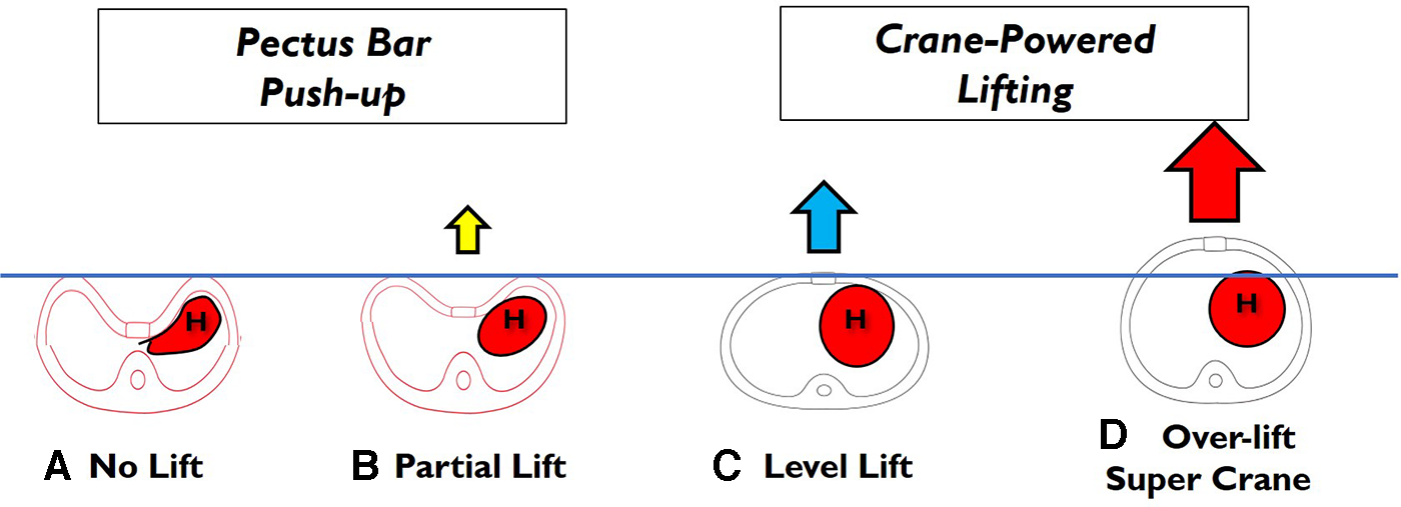
Totally craned approach. (**A**) No lift of the depressed sternum: the chest wall elevation relies solely on the pectus bar lever action. (**B**) Partial lift of the depressed sternum: better visualization but full lifting needs to be achieved through the pectus bar lever action. (**C**) The sternum is lifted to the target level using machine lifting, eliminating the need for pectus bar lever action. (**D**) The sternum is over-lifted beyond the target level, and the chest wall elevation is entirely achieved through machine lifting, without requiring pectus bar lever action.

In contrast, the crane-powered approach initiates chest wall pre-elevation to a level well above the target. Subsequently, the pectus bars are positioned accurately with minimal effort, and the chest wall is gently lowered onto the bars by releasing the crane elevation (Figure 4). By pre-lifting the chest wall above the desired level beforehand, the procedure becomes easier, safer, and eliminates the challenges associated with the traditional pectus bar lifting technique. This innovative approach aims to enhance the safety and effectiveness of pectus deformity repairs by addressing the limitations of the conventional method (Figure 5).

The crane-powered approach enables us to accurately anticipate the extent of chest wall elevation in each individual case. Our method entails elevating the chest wall significantly above the intended target level, thereby facilitating the precise placement of pectus bars at the appropriate heights. Subsequently, we gently lower the chest wall onto the bars by releasing the crane. Should the chest wall elevation not align with our initial expectations, we can easily retract the bars and make necessary adjustments to achieve the desired convexity, ensuring that over-correction or under-correction of the deformity is avoided. This approach provides a superior degree of control and precision, facilitating the attainment of optimal results.

### Refinement of the crane system

**Figure 6:**
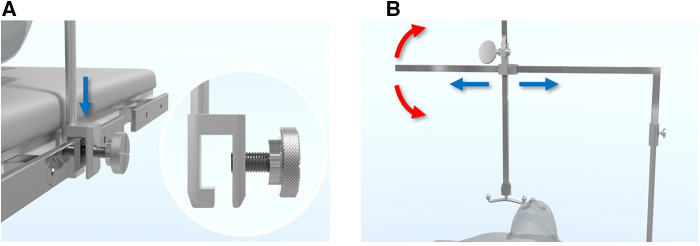
Easy crane system for pectus excavatum repair. (**A**) The Easy Crane system is effortlessly mounted on the operating table by sliding it down from above to the side rail, securely fitting into the slot of the crane pole (blue arrow). (**B**) The crane's arm is straightforward to assemble and can be easily adjusted for the desired lifting location by swinging around (red arrows) and altering the arm's length (blue arrows).

The initial crane was simply sternal wire attached to any available retractors. The author finally designed a pectus surgery specific crane system, the Easy Crane (Primemed, Seoul, South Korea). The Easy Crane is designed to be attached to any position at the side bar of the operating table as well as to any point of the chest wall to accommodate the location of lifting. The installation and removal is per demand and easy to slip on to the rails without hard locking, which makes it liberally applicable at any time during the operation (Figure 6).

### Introduction of the screw-crane system

**Figure 7:**
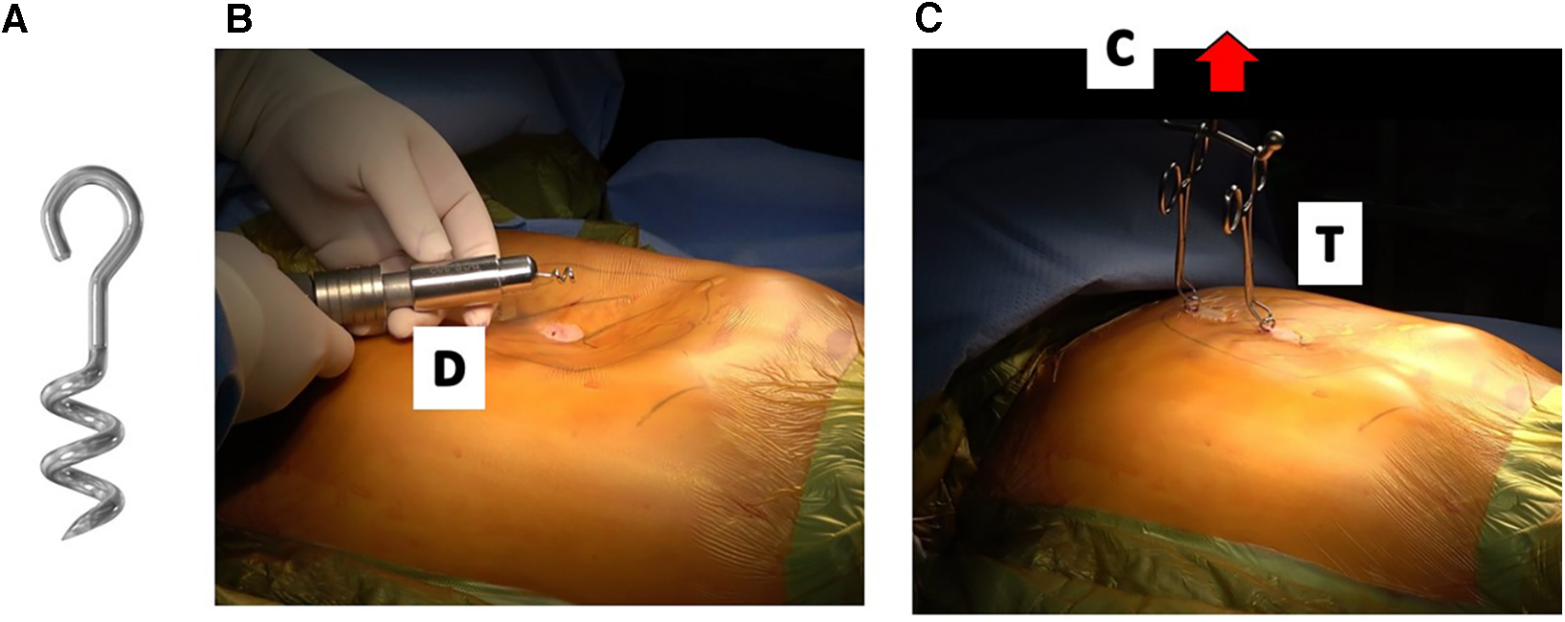
Sternal elevation with corkscrew–type crane (2019 – present). (**A**) Utilization of corkscrew-type screws (2nd Generation) for crane elevation, ensuring secure stability with heavy lifting. (**B**) Minimal scarring technique: utilizing a skin nick for screw driving into the sternal bony matrix, resulting in strong elevation with minimal visible scarring. D: screw driver. (**C**) Crane-assisted elevation: achieving optimal chest wall correction by lifting the sternum to full level (red arrow), flexibility for implementing multiple screws as required. Towel clips facilitate procedure by connecting screws to the crane. C: Easy crane, T: towel clips.

Previously, the crane technique involved sternal wire stitching at specific points of the sternum, depending on the repair targets. While sternal stitching was a convenient and safe approach, blind sternal suturing posed discomfort for some surgeons. To address these concerns and enhance the technique's universal adaptability, the author developed the screw-crane system. Initially, the design with ordinary threads had limitations, as it could be pulled out with heavy force. As a result, a corkscrew-style screw was developed, which proved more effective at supporting the heavy sternum during complete elevation while leaving minimal scarring (21) (Figure 7).

We employ two different screw lengths, 10 mm and 15 mm, in the spiraled section, based on the sternal thickness for anchoring. Prior to the procedure, we establish the appropriate screw driving depth by pre-determining the sternal thickness through CT scan measurements. The choice of screw size is customized for each patient based on these measurements.

## Conclusion

The crane-powered pectus excavatum repair demonstrated favorable outcomes with low major complication rates and zero occurrences of bar displacement. These results highlight the safety and effectiveness of this innovative surgical approach for the correction of pectus excavatum deformities.

The Crane-powered Pectus Surgery, as a component of the x surgery, marks a momentous advancement in pectus excavatum correction by harnessing the power of a crane machine to elevate the chest wall. By surmounting the challenges encountered in the conventional Nuss procedure, this novel approach ensures safer and more effective outcomes for patients, heralding a new era in pectus excavatum repair. The NeoPectus surgery, with its comprehensive and precise chest wall remodeling, holds immense promise for transforming the concept of pectus excavatum repair and improving the overall quality of patient care in this field.

## Data Availability

The raw data supporting the conclusions of this article will be made available by the authors, without undue reservation.
